# Large amplitude vibrations of imperfect spider web structures

**DOI:** 10.1038/s41598-020-76269-x

**Published:** 2020-11-05

**Authors:** Sakdirat Kaewunruen, Chayut Ngamkhanong, Simiao Xu

**Affiliations:** 1 Department of Civil Engineering, School of Engineering, University of Birmingham, Birmingham, B15 2TT UK; 2 Shanghai Posts and Telecommunications Designing Consulting Institute Co., Ltd., Shanghai, China

**Keywords:** Computational biology and bioinformatics, Engineering, Mathematics and computing

## Abstract

Due to the high-efficiency energy absorption and high-tension strength material properties of spider silk, many researchers have studied the mechanical properties and microstructure of the spider web. The concept of spider web structure has been recognized to be adopted for structural engineering aspect. The structure of spider web and its material properties have been studied for decades. However, the fundamental free vibration mode shapes and their corresponding frequencies have never been fully investigated. This study investigates the nonlinear characteristics in the large-amplitude free vibration of imperfect spider web structures using finite element analysis. The spider web applies the concept of elastic cables taking only axial deformation into account. The finite element models of a spider web considering geometric nonlinearities are employed. It should be noted that spider web could experience large deformation when the spider uses its silk to catch prey. This research aims at analyzing the linear and geometric nonlinear behaviour of imperfect spider web structure. Four different types of imperfect spider web: spiral imperfect spider web, radial imperfect spider web, central imperfect spider web, and circular rings imperfect spider web, are considered. It is found that pretension in spider silk plays a significant role in nonlinear vibration characteristics of the spider web. Moreover, the radial thread damaged tends to have a greater effect on structural free vibration of spider web in comparison with other imperfections. The outcome will help a structural engineer to adapt the concept of spider web, its properties, and damage patterns for any larger structures.

## Introduction

Recently, spider web structure has been gradually recognized as outstanding silk in term of performance among most manmade fibres. As one of the bio-inspired materials, there have been the interests in adopting the spider web engineering properties in defence technology and medical applications, including microscopes, telescope and bomb guiding systems^1^. Due to extensional properties when prey or external forcing caused structural displacement, spider web structure can absorb the kinetic energy and dissipate about 70% of the converted energy to prevent the structural function, therefore the ability to dissipate energy on account of high stress–strain rates applied to body armour system for ballistic protection^2^. On the other way, silk materials can be made into degradable suture to reduce rejection and help blood clot in an aspect of biomedical application. It is noted that the spider web structure is naturally constructed as pre-stressed systems called “Tensegrity (Tensional integrity)” structures^3^. The pretension in spider string can prevent the large deformation or slack of the string resulting in preventing the nonlinear geometry of string.

More importantly, spider silk structure is able to enhance the strength and stiffness in fibres^4^, the common examples of silk structural application are adopted for elevator ropes, and high strength cable. Although biomimetic material and web structure have been used in many fields, more superior performances of spider web are worth to explore. The stress–strain behaviour and nanostructure of different types of spider silks have been previously discussed involving their engineering properties^5,6^. The mechanical properties of spider silk have been discussed^7–9^. The tensile stress–strain curve of spider silk has been studied and compared with other fibres^10^. It is found that its curve tends to be quite similar to that of an elastomer. Note that this rubber like stress–strain curve is a very common feature characterizing spider drag-line silk. On the other hand, the mechanical properties of spider web structure were mainly effected by hierarchical assembly into fibres of spider silk^11^. The semi-amorphous domain and beta-sheet nanocrystal are the basic nanostructures that decide the material properties of spider silk^12–14^. It should be noted that the toughness and stiffness of spider silk are likely to be higher in comparison to other man-made fibres^3^. It is noted that there are two main thread elements in the spider web structure: spiral thread and radial thread based on geometry. The functions of each thread have been discussed previously^10^. The load is separately applied on both radial and spiral treads. It is found that structural performance is dominated by the radial thread while the spiral tread acts as a non-structural member higher force are required to break the radial thread.

Noting that the spiral threads become nearly fully aligned with the loading direction prior to failure (with an angle of 10°) as the thread under loading reaches 4.5 times of its initial length and the two points connecting to the rest of the web structure get closer under large deformation^15^. Hence, spider web element is more likely to be modelled as a string instead of beam or truss. Spider web could experience large deformation due to wind load or prey trapping^15^. It should be noted that the spider web should be able to trap the large prey. This effect allows the web to absorb high kinetic impacts that would otherwise cause an extension above the elastic range and therefore destroy the web. Nevertheless, the studies of the structural behaviour regarding the vibration characteristics of spider webs have not been fully investigated. Generally, the natural frequencies and the corresponding mode shapes are independent of vibration amplitude according to linear theory. However, if the amplitude of mode shape becomes large, this results in significant nonlinear behaviours, the spider silk nonlinear dynamics have to be analysed^16^. The dynamic responses of the spider web under shock load have been performed assuming the web structure as SDOF system. The free vibration responses subjected to shock load could extract the stiffness and damping of the spider web. Comparing to the experimental result, it is found that nonlinear stiffness model consisting of dry friction, coulomb damping and nonlinear stiffness, can better represent the behaviour of spider web under large deformation^17^. As for the energy dissipation of the spider web, the dynamic nonlinear model more accurately describes the behaviour of structure. Therefore, the reasonable hypothesis can be obtained, nonlinear mode similarly applies on large amplitude free vibration of spider webs rather than the linear mode. It is noted that the stiffness has to be updated if the structure is subjected to large deformation so that geometric nonlinear analysis is essential. Even though the vibration of spider web has been investigated^18,19^, the study of large vibration of imperfect spider web has never been investigated in the past. In this study, the three-dimensional imperfect spider web structures have been developed based on different the cable configurations and defects. The linear and nonlinear free vibration behaviours are analysed to obtain the fundamental mode shapes and their corresponding frequencies. The influences of pretension in the spider web are investigated in the nonlinear analysis. The nonlinear geometry is taken into account in nonlinear analysis due to the large deformation of spider web.

## Methods

### Equations of motion

The concept of cable structure can be applied to spider silk since the structure of spider silk is naturally a special class of prestressed system called tensional integrity (tensegrity). Hence, to investigate the vibration responses of spider silk, the strain energy method considering only axial deformation is applied. The configuration states of spider silk can be distanced into three states: unstretched state, equilibrium state, and dynamic or displaced state. The first stage is the initial unstretched state *dS* at *(x,y)*. After tension applied, the cable is elongated and the structure moves to the equilibrium state at *dS*_*0*_ at *(x*_*0*_*,y*_*0*_*)* which is considered as the initial configuration for dynamic state On account of free vibration at dynamic or displaced state $d\overline{s}$, spider silk displaces to the position of *(x*_*0*_ + *u, y*_*0*_ + *v, w)*, in which *u*, *v* and *w* are the hypothetic displacements in the direction of *x*, *y* and *z*-axis, respectively^20,21^ . The length of $d{s}_{0}$ is an infinitesimal spider silk element, the equilibrium state can be represented as

1 $${d{s}_{0}}^{2}={d{x}_{0}}^{2}+{d{y}_{0}}^{2} \mathrm{or }d{s}_{0}=\sqrt{1+{\dot{y}}_{0}^{2}}d{x}_{0}$$

Similarly, the equations of unstretched state $ds$ and displaced state $d\overline{s}$ can be written as

2 $$ds=\frac{\sqrt{1+{\dot{y}}_{0}^{2}}}{1+{\varepsilon }_{0}}d{x}_{0}$$

3 $$d\overline{s}=\sqrt{{(1+\dot{u})}^{2}+{({\dot{y}}_{0}+\dot{v})}^{2}+\dot{{w}^{2}}}d{x}_{0}$$

where $$\dot{u}=\frac{du}{dx}$$ , $$\dot{v}=\frac{dv}{dx}$$ , $$\dot{w}=\frac{dw}{dx}$$

Total strain due to stretching of the cable can be derived at the displaced state by

4 $$\overline{\varepsilon }=\frac{d\overline{s}-ds}{ds}=\frac{(1+{\varepsilon }_{0})}{\sqrt{1+{\dot{y}}_{0}^{2}}}\sqrt{{(1+\dot{u})}^{2}+{({\dot{y}}_{0}+\dot{v})}^{2}+\dot{{w}^{2}}}-1 $$

where ${\varepsilon }_{0}$ is the initial static strain.

The virtual strain energy equation taking dissipated energy at the displaced state due to the stretching of the cable can be written as

5 $$\delta U={\int }_{0}^{s}EA\stackrel{-}{\varepsilon }\left(\delta \stackrel{-}{\varepsilon }\right)ds$$

where E is the elastic modulus, A is the crosssectional area.

Using Eqs. (2) and (4), Eq. (5) can be rewritten as

6 $$\delta U={\int }_{0}^{{X}_{H}}\left(\left(\frac{EA\left(1+{\varepsilon }_{0}\right)\dot{u}+EA}{\sqrt{1+{\dot{y}}_{0}^{2}}}-\frac{EA\left(1+\dot{u}\right)}{\sqrt{{\left(1+\dot{u}\right)}^{2}+{\left({\dot{y}}_{0}+\dot{v}\right)}^{2}+\dot{{w}^{2}}}}\right)\delta u{^{\prime}}+\left(\frac{EA\left(1+{\varepsilon }_{0}\right)\dot{v}+EA{\dot{y}}_{0}}{\sqrt{1+{\dot{y}}_{0}^{2}}}-\frac{EA\left({\dot{y}}_{0}+\dot{v}\right)}{\sqrt{{\left(1+\dot{u}\right)}^{2}+{\left({\dot{y}}_{0}+\dot{v}\right)}^{2}+\dot{{w}^{2}}}}\right)\delta v{^{\prime}}+\left(\frac{EA\left(1+{\varepsilon }_{0}\right)\left(\dot{w}\right)}{\sqrt{1+{\dot{y}}_{0}^{2}}}-\frac{EA\dot{w}}{\sqrt{{\left(1+\dot{u}\right)}^{2}+{\left({\dot{y}}_{0}+\dot{v}\right)}^{2}+\dot{{w}^{2}}}}\right)\delta w{^{\prime}}\right)d{x}_{0}$$

It is noted that tension at the equilibrium can be represented as $T=EA{\varepsilon }_{0}$. It can be substituted to Eq. (6) in order evaluate its effect on virtual strain energy.

The virtual work done by inertial force can be derived by

7 $${\delta W}_{i}=-{\int }_{0}^{l}\frac{m\sqrt{1+{\dot{y}}_{0}^{2}}}{g\left(1+{\varepsilon }_{0}\right)}(\ddot{u}\delta u+\ddot{v}\delta v+\ddot{w}\delta w)d{x}_{0}$$

Total energy (${\delta  \Pi  })$ can be derived by^20^

8 $$\delta  \Pi  =\delta U-\delta {W}_{i}=0$$

where ${\delta  \Pi  }$ is total energy, $\mathrm{\delta U}$ is a variation of strain energy, $\updelta {W}_{i}$ is virtual work done due to initial force.

The pinned support conditions are applied at both ends of the silk member so that the boundary conditions are *δu* = *δv* = *δw* = 0 when *x*_*0*_ = 0 and *x*_*0*_ = *l.* After substituting the boundary conditions and equilibrium conditions ($\updelta \dot{u}=\updelta \dot{v}=\updelta \dot{w}=\updelta \ddot{u}=\updelta \ddot{v}=\updelta \ddot{w}=0$) into the total energy equation, the governing equations of motions can be expressed as shown in Eqs. (9)–(11). These equations are used to analyse the three-dimensional large amplitude free vibration with the undamped system.

9 $${\left[\frac{EA\left(1+{\varepsilon }_{0}\right)\dot{u}+EA}{\sqrt{1+{\dot{y}}_{0}^{2}}}-\frac{EA\left(1+\dot{u}\right)}{\sqrt{{\left(1+\dot{u}\right)}^{2}+{\left({\dot{y}}_{0}+\dot{v}\right)}^{2}+\dot{{w}^{2}}}}\right]}^{^{\prime}}-\frac{m\sqrt{1+{\dot{y}}_{0}^{2}}}{g\left(1+{\varepsilon }_{0}\right)}\ddot{u}=0$$

10 $${\left[\frac{EA\left(1+{\varepsilon }_{0}\right)\dot{v}+EA{\dot{y}}_{0}}{\sqrt{1+{\dot{y}}_{0}^{2}}}-\frac{EA\left({\dot{y}}_{0}+\dot{v}\right)}{\sqrt{{\left(1+\dot{u}\right)}^{2}+{\left({\dot{y}}_{0}+\dot{v}\right)}^{2}+\dot{{w}^{2}}}}\right]}^{^{\prime}}-\frac{m\sqrt{1+{\dot{y}}_{0}^{2}}}{g\left(1+{\varepsilon }_{0}\right)}\ddot{v}=0$$

11 $${\left[\frac{EA(1+{\varepsilon }_{0})(\dot{w})}{\sqrt{1+{\dot{y}}_{0}^{2}}}-\frac{EA\dot{w}}{\sqrt{{\left(1+\dot{u}\right)}^{2}+{\left({\dot{y}}_{0}+\dot{v}\right)}^{2}+\dot{{w}^{2}}}}\right]}^{^{\prime}}-\frac{m\sqrt{1+{\dot{y}}_{0}^{2}}}{g\left(1+{\varepsilon }_{0}\right)}\ddot{w}=0$$

By substituting tension $T=EA{\varepsilon }_{0}$ to Eqs. (9)-(11), the gov equations can be rewritten as shown in Eqs. (12)-(14).

12 $${\left[\frac{T\dot{u}+EA\left(1+\dot{u}\right)}{\sqrt{1+{\dot{y}}_{0}^{2}}}-\frac{EA\left(1+\dot{u}\right)}{\sqrt{{\left(1+\dot{u}\right)}^{2}+{\left({\dot{y}}_{0}+\dot{v}\right)}^{2}+\dot{{w}^{2}}}}\right]}^{^{\prime}}-\frac{m\sqrt{1+{\dot{y}}_{0}^{2}}}{g\left(1+{\varepsilon }_{0}\right)}\ddot{u}=0$$

13 $${\left[\frac{T\dot{v}+EA\left({\dot{y}}_{0}+\dot{v}\right)}{\sqrt{1+{\dot{y}}_{0}^{2}}}-\frac{EA\left({\dot{y}}_{0}+\dot{v}\right)}{\sqrt{{\left(1+\dot{u}\right)}^{2}+{\left({\dot{y}}_{0}+\dot{v}\right)}^{2}+\dot{{w}^{2}}}}\right]}^{^{\prime}}-\frac{m\sqrt{1+{\dot{y}}_{0}^{2}}}{g\left(1+{\varepsilon }_{0}\right)}\ddot{v}=0$$

14 $${\left[\frac{T\dot{w}+EA\dot{w}}{\sqrt{1+{\dot{y}}_{0}^{2}}}-\frac{EA\dot{w}}{\sqrt{{\left(1+\dot{u}\right)}^{2}+{\left({\dot{y}}_{0}+\dot{v}\right)}^{2}+\dot{{w}^{2}}}}\right]}^{^{\prime}}-\frac{m\sqrt{1+{\dot{y}}_{0}^{2}}}{g\left(1+{\varepsilon }_{0}\right)}\ddot{w}=0$$

### Finite element modelling

#### Material and boundary condition

**Figure 1:**
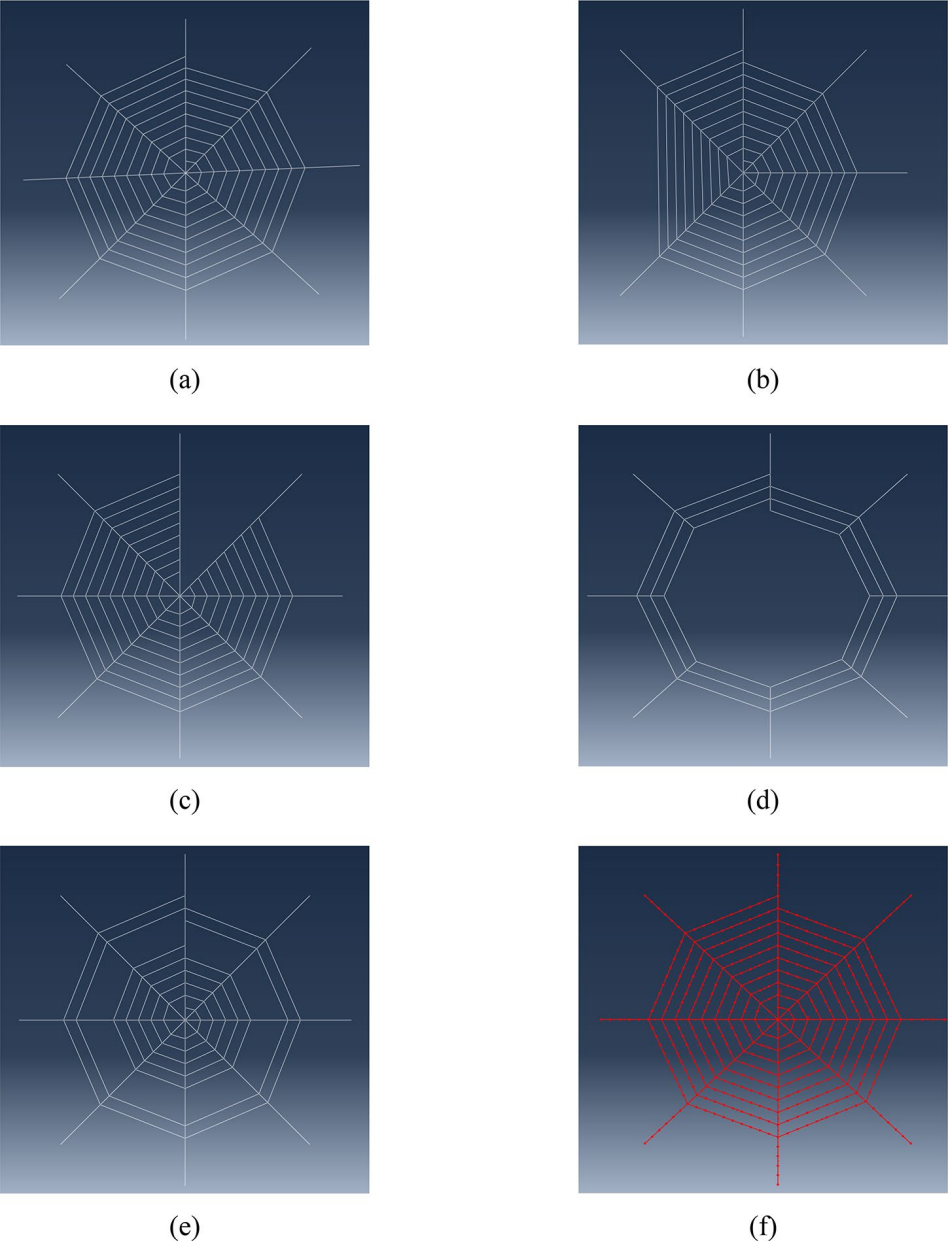
**(a)** Spider web, **(b)** radial threads imperfect spider web, **(c)** spiral threads imperfect spider web, **(d)** central imperfect spider web, **(e)** circular rings imperfect spider web, **(f)** mesh generation.

Recently, the mechanical properties of spider silk have been investigated for different species. This study considers a dragline silk of *Argiope aurantia* Spider Silk as the structural member in the numerical simulations since this is the strongest type of spider silk, among others. Generally, spiral threads are made of viscid silk which has lower strength but higher extensibility than dragline silk and this combination optimise the function of a spider web. However, in this paper, it focusses on the structure behaviour itself, therefore, the same material properties have been applied to both radial and spiral thread to provide better structural behaviour. It should be noted that the higher elastic modulus of web element can build stiffer structure resulting in higher vibration frequencies. According to the investigation by^9^, most of the spider silks have a circular fibre cross-section. The engineering properties of *Argiope aurantia* spider silk are as follows: density = 1098 kg/m^3^, tensile modulus, *E*_*L*_ = 0.003 GPa, Poisson's Ratio (*v*) = 0.49. The stress–strain curve consists of three distinct regions. Region I (0–5%) is characterized by a high initial modulus of 34 GPa representing the elastic phase. After reaching the plastic phase, there are two regions: region II (5–21%) shows a pseudo yield point at 5% before strain hardening to a maximum modulus of 22 GPa at 22% elongation, and region III (21–36%) exhibits a gradual reduction of modulus until reaching failure strength of 1.75 GPa at 36% elongation. The spider web model is developed on the 2-D plane (X and Y axes) while vibrating in a third axis (Z axis). The model geometry considered is referred to ^17^ and it is scaled up doubly as the largest spider web diameter found in reality can be between 800 and 1000 mm. The total size of the spider web is approximately 800 mm × 800 mm with the diameter of 4 μm. The length of each radial thread is 400 mm and the interval between spiral thread is approximately 30 mm. The boundary conditions have exhibited the characteristic of spider web anchorages. The node ends are constrained translationally while the nodes are allowed to rotate freely. The perfect spider web is presented in Fig. 1a. Moreover, four different patterns of imperfect spider webs (spiral thread imperfection, radial thread imperfection, central imperfection and circular ring imperfection) are built as shown in Fig. 1b–e, respectively. Each pattern presents different damage levels. This paper analyses ten patterns of spiral thread imperfect spider web, 9 patterns of radial thread imperfect spider web, nine patterns of central imperfect spider web, and 5 patterns of circular ring imperfect spider web. As for modelling, mesh convergence analysis is considered by resizing the element size from between 0.01 and 0.1 m to generate a proper mesh size and accurately compute the FEM results. It is noted that the automated meshing by ABAQUS can clearly provide the proper mesh size as presented in Fig. 1f. Material, geometric, as well as nonlinearity, are included in the analysis algorithm. The linear model extracts natural frequencies due to free vibration. It should be noted that linear analysis can properly analyse the structural responses restricted to small deformation so that the small deformation and constant stiffness are the fundamental assumptions of linear analysis. However, in fact, the stiffness may change when the structure is subjected to large deformation. Hence, it is necessary to consider the nonlinear effects when the structure experiences large amplitude vibration. This paper studies the free vibration responses of spider web structure. The analysis methods are divided into two parts. The linear analysis is firstly studied assuming that stiffness change is negligible. In the second part, the large amplitude free vibration is considered in geometrically nonlinear analysis and the results are compared with those obtained from linear analysis.

#### Model validation

**Figure 2:**
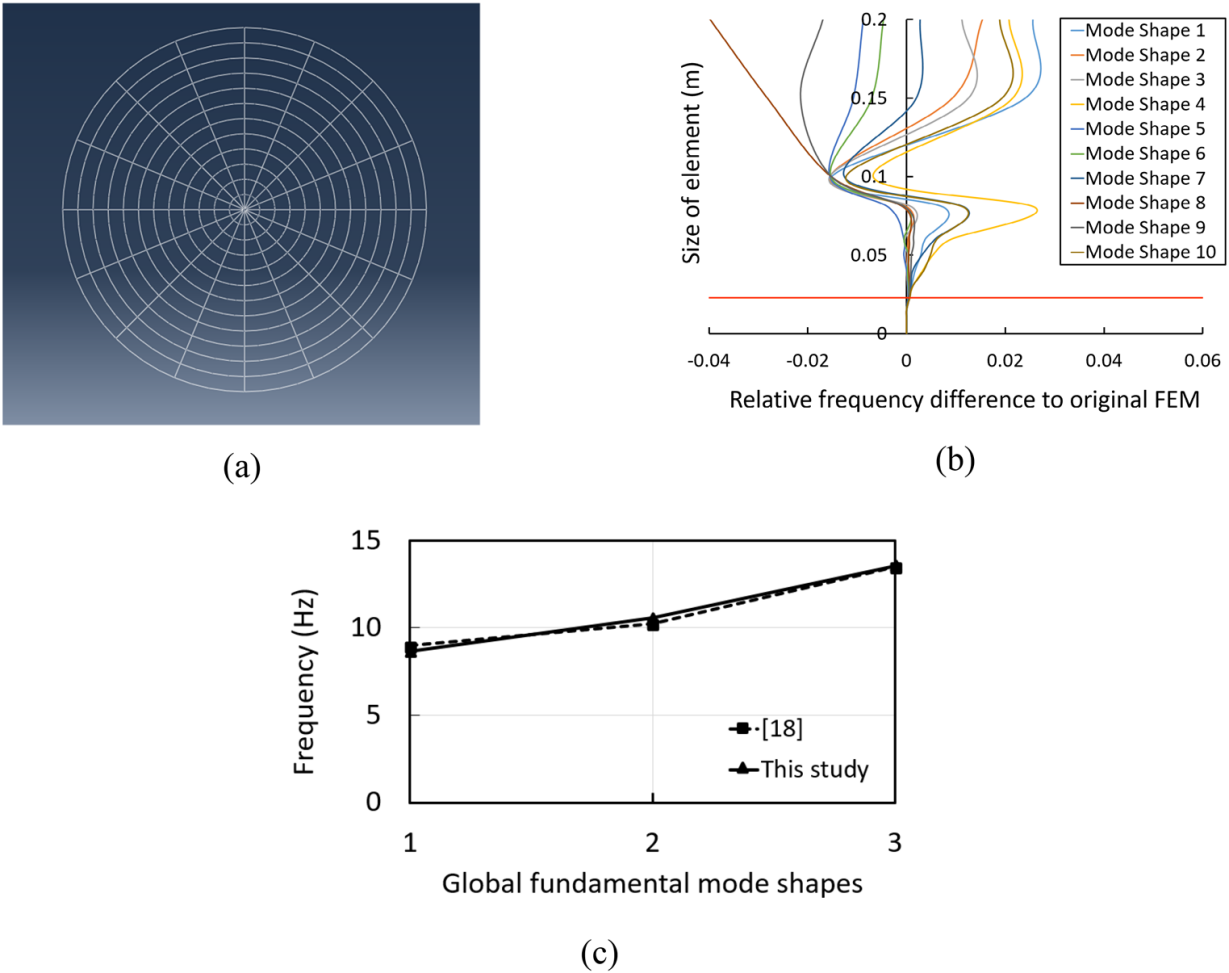
Model validation: **(a)** spider web model in ABAQUS, **(b)** mesh convergence analysis, **(c)** natural frequencies comparison with previous study.

To analysis the free vibration characteristic of spider web including mode shapes and corresponding frequencies, the linear eigenvalue is firstly used. Due to the characteristic of actual spider webs that are very complex and have different shapes depending on their types, the accuracy of measurement requires a high level of model accuracy. Hence, the spider web is compared with the previous study which has been validated. The model is made in accordance with the artificial spider web using nylon and rubber properties^18^, as presented in Fig. 2a. It should be noted that the validation model is much smaller than the actual spider web. The model consists of 16 nylon radial threads and 12 rubber spiral threads with the spacing of 15 mm. The spacing between the rubber spiral threads is 15 mm. The diameter of spider silk and its elastic modulus have been adopted to 500-800 μm and 3 GPa, respectively, to match the relevant. Mesh convergence analysis has been done and it is found that proper mesh size is 23 mm which can be generated automatically in ABAQUS (Fig. 2b). The results show that the differences in frequencies between the previous study and this study are less than 4% as seen in Fig. 2c.

## Results and discussions

### Progressive failure linear analysis

**Figure 3:**
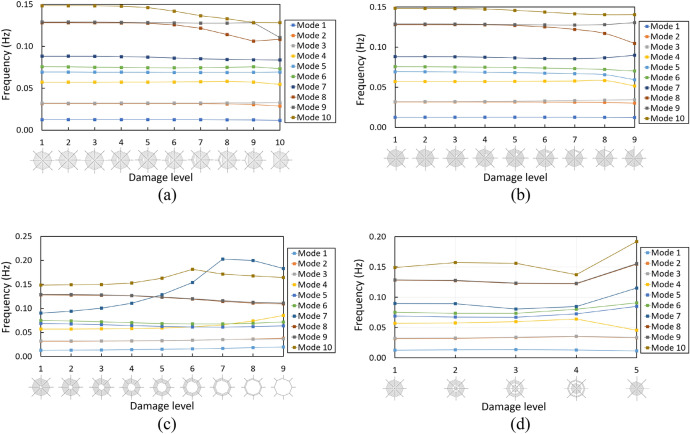
Natural frequencies of imperfect spider web; **(a)** radial threads imperfect spider web, **(b)** spiral threads imperfect spider web, **(c)** central imperfect spider web, **(d)** circular rings imperfect spider web.

The damaged spider web structures are analysed considering linear finite element model to obtain the natural frequencies and mode shapes. In this part, ten patterns exhibit the different damage levels of radial defective spider webs (Fig. 3a). There is no significant change in natural frequencies from mode 1 to mode 7 when the spider web is damaged. These results illustrate that the series which from mode 1 to mode 7 have been hardly affected due to the increased defect level of radial thread. Until mode 8, the frequencies have been a significant drop with high-level radial imperfect (8, 9 and 10). It can be concluded that the radial defect is likely to affect the free vibration at higher modes. As for the spiral imperfect spider web in Fig. 3b, the innermost of spiral thread is firstly removed, then outer spider threads are removed thread by thread. For the series of mode shapes 1–7, the removal of spiral threads has slight effects in natural frequencies, but the natural frequencies have no specific change. For mode 8, the natural frequencies continuously decreased with the damaged levels 7, 8 and 9. In case of central imperfect spider web, both spiral and radial threads are removed altogether as shown in Fig. 3c. The damaged zone is gradually expanded from the inner loop to the outer loop. The effects of centre-damaged impacts turn out to be small for the modes 5, 6, 8 and 9 as well. For lower modes, mode 2 is crossed over by mode 3 from the pattern of damage level 4 to damage level 5. Besides, it is worth noting that the natural frequencies of modes 4, 7 and 10 have significant changes under the condition of central damaged. In particular, the natural frequencies of modes 4 and 7 have a big jump and cross over with each higher mode. The frequencies of mode 4 have a continuous enhancement at the beginning from damage level 5, and finally, the natural frequency of mode 4 is higher than modes 5 and 6. By contrast, the natural frequencies of mode 7, which cross over with modes 8, 9 and 10, sharply rise until it reaches the highest at the damage level 7. Different from Case 2, Case 4 removes rings of spiral threads as shown in Fig. 3d. Two rings of spiral threads are gradually removed from damage level 1 to damage level 5. It should be noted that this case can be compared to central imperfect. However, the radial silk is kept with no damage as radial silk has been known as a structural member. From Fig. 3, it is confirmed that radial thread imperfection causes significant effects rather than spiral threads. To be concluded, radial imperfect and central imperfect have significant influences when the spider web structures are gradually destroyed. Spiral and circular ring imperfections have relatively small effects on the natural frequencies in comparison to radial and central imperfections. This is because the radial threads play an important role as a structural member for the spider web structure. Besides, the natural frequencies and the corresponding mode shapes are significantly changed in high-damage level especially in higher modes of vibration. It illustrates that the imperfect spider web structure is highly sensitive to geometric imperfections.

### Geometric nonlinear behaviour of spider web

**Figure 4:**
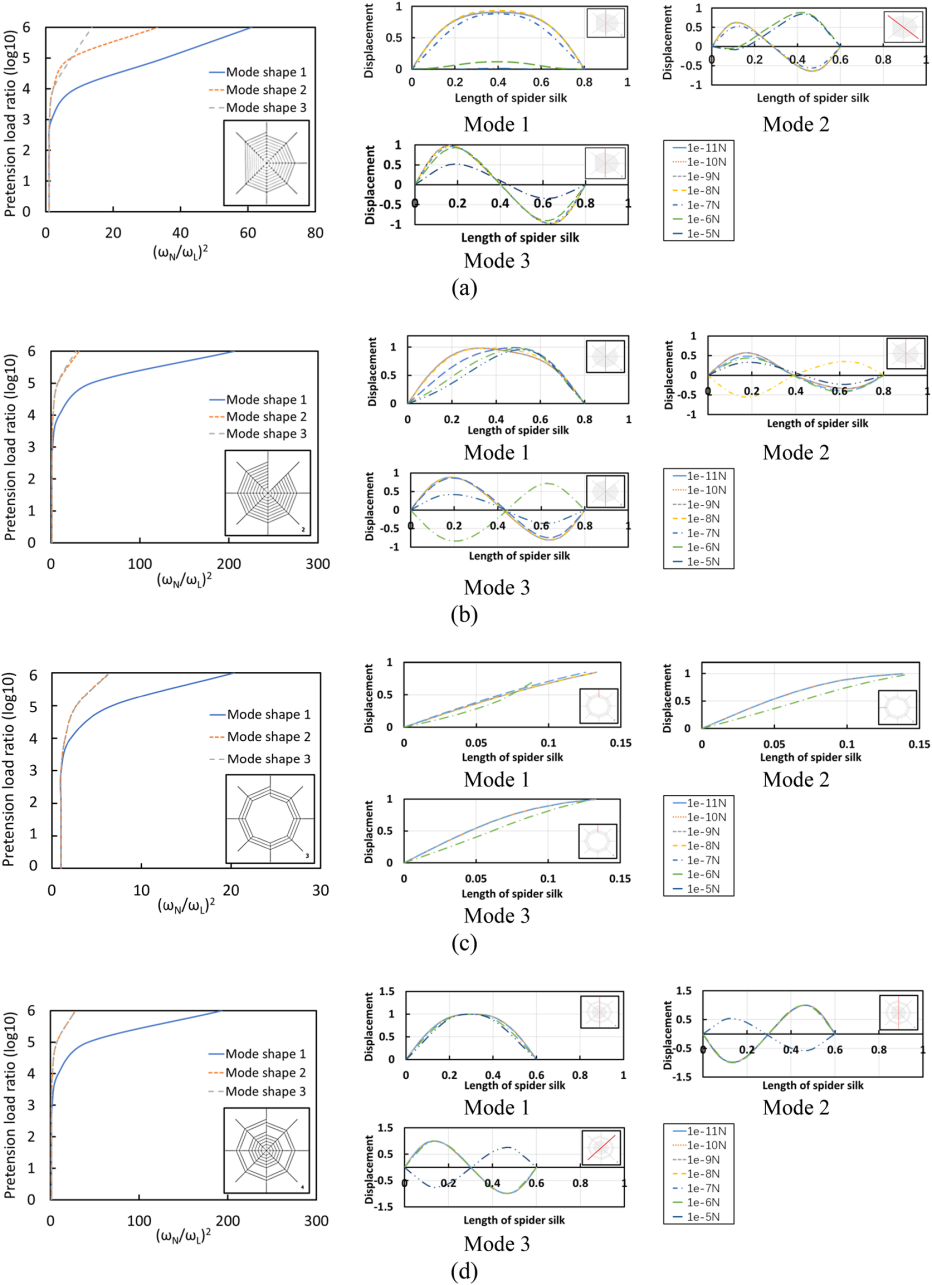
Geometric nonlinear behaviour of imperfect spider web considering pretension load: **(a)** radial imperfect spider web, **(b)** spiral imperfect spider web, **(c) **central imperfect spider web, **(d)** circular ring imperfect spider web.

This part investigates the significance of geometrically nonlinear analysis on the progressive failure response of spider web structure. During the process via iterative solution, the stiffness is updated due to the large deformation. The nonlinear geometry is taken into account considering the effect of pretension force. It should be noted that the pretension force is one of the key parameters affecting the performance of the spider web to catch the prey. In this study, the pretension force inducing a large amplitude vibration is varied from 10^−11^ N to 10^−5^ N as the initial condition. The nonlinear frequencies are compared with the corresponding linear frequencies that have been analysed previously. The results are presented in terms of pretension load ratio (pretension force/10^–11^) and frequency ratio (Nonlinear frequency/linear frequency) as seen in Fig. 4. It should be noted that the pretension force significantly influences the mode shapes, especially in the higher frequency so that it is difficult to identify the corresponding frequencies. Hence, only the first three fundamental modes are extracted.

**Figure 5:**
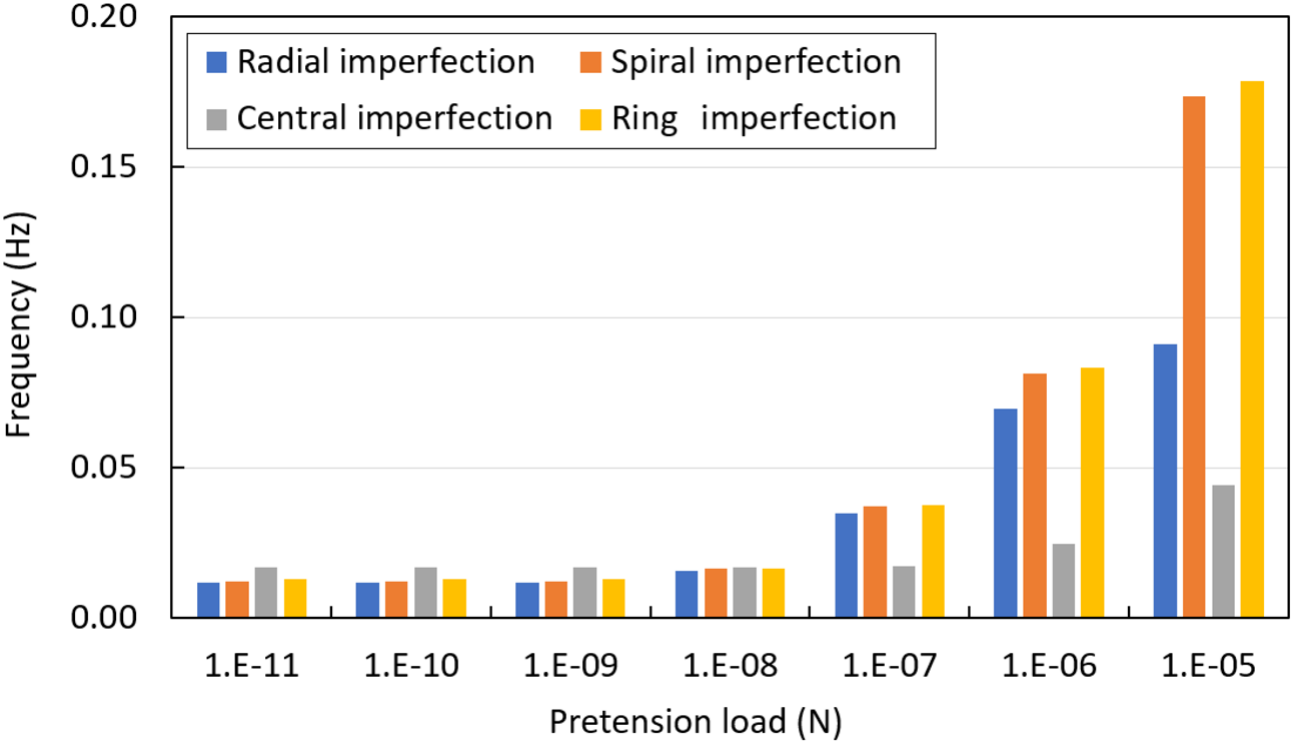
Natural frequency of spider web considering pretension load.

Geometric nonlinear effects become prominent when the structure is subjected to large displacement. According to the obtained results, the nonlinear free vibration which is caused by pretension load is discussed. In brief, four different types of imperfect spider web indicate different levels of the responses. As the initial conditions, pretension leads to an increase in flexural stiffness. Although each mode shape has different natural frequencies compared to others, the trends of nonlinear frequencies behaviour tend to be similar as the spider web is in the hardening stage when pretension load is 10^−7^ N. Figure 5 illustrates the effect of pretension load on mode 1, the natural frequencies are increased with the increase in pretension load. It is clear that geometric nonlinearity has a strong influence on the spiral and circular ring imperfect structures, while radial and central imperfections are affected slightly. It illustrates that spiral threads are more sensitive than radial threads to the variation of pretension load.

## Conclusions

This study investigates the large amplitude free vibration behaviour of imperfect spider web. The natural frequencies and corresponding mode shapes are obtained by finite element analysis software ABAQUS. The results are divided into two parts: progressive failure linear analysis and geometric nonlinear analysis. The natural frequencies indicate nonlinear behaviour with different patterns. Spider web is a sensitive structure to geometric imperfection since crossover frequencies are observed especially when the structure is gradually damaged to the high level. This can be observed on radial imperfect and central imperfect structures that have significant influences when the spider web structures are gradually destroyed. As for geometric nonlinear behaviour, pretension load leads to hardening stage due to the large free vibration. Geometric nonlinearity has a strong influence on spider web structure especially the spiral imperfect and circular rings imperfect structures. It illustrates that pretension force can result in the nonlinear behaviour on spider free vibration.
